# High-Throughput Identification of Adaptive Mutations in Experimentally Evolved Yeast Populations

**DOI:** 10.1371/journal.pgen.1006339

**Published:** 2016-10-11

**Authors:** Celia Payen, Anna B. Sunshine, Giang T. Ong, Jamie L. Pogachar, Wei Zhao, Maitreya J. Dunham

**Affiliations:** 1 Department of Genome Sciences, University of Washington, Seattle, Washington, United States of America; 2 Department of Biostatistics, University of Washington, Seattle, Washington, United States of America; Stanford University School of Medicine, UNITED STATES

## Abstract

High-throughput sequencing has enabled genetic screens that can rapidly identify mutations that occur during experimental evolution. The presence of a mutation in an evolved lineage does not, however, constitute proof that the mutation is adaptive, given the well-known and widespread phenomenon of genetic hitchhiking, in which a non-adaptive or even detrimental mutation can co-occur in a genome with a beneficial mutation and the combined genotype is carried to high frequency by selection. We approximated the spectrum of possible beneficial mutations in *Saccharomyces cerevisiae* using sets of single-gene deletions and amplifications of almost all the genes in the *S*. *cerevisiae* genome. We determined the fitness effects of each mutation in three different nutrient-limited conditions using pooled competitions followed by barcode sequencing. Although most of the mutations were neutral or deleterious, ~500 of them increased fitness. We then compared those results to the mutations that actually occurred during experimental evolution in the same three nutrient-limited conditions. On average, ~35% of the mutations that occurred during experimental evolution were predicted by the systematic screen to be beneficial. We found that the distribution of fitness effects depended on the selective conditions. In the phosphate-limited and glucose-limited conditions, a large number of beneficial mutations of nearly equivalent, small effects drove the fitness increases. In the sulfate-limited condition, one type of mutation, the amplification of the high-affinity sulfate transporter, dominated. In the absence of that mutation, evolution in the sulfate-limited condition involved mutations in other genes that were not observed previously—but were predicted by the systematic screen. Thus, gross functional screens have the potential to predict and identify adaptive mutations that occur during experimental evolution.

## Author Summary

Experimental evolution allows us to observe evolution in real time. New advances in genome sequencing make it trivial to discover the mutations that have arisen in evolved cultures; however, linking those mutations to particular adaptive traits remains difficult. We evaluated the fitness impacts of thousands of single-gene losses and amplifications in yeast. We discovered that only a fraction of the hundreds of possible beneficial mutations were actually detected in evolution experiments performed previously. Our results provide evidence that 35% of the mutations identified in experimentally evolved populations are advantageous and that the distribution of beneficial fitness effects depends on the genetic background and the selective conditions. Furthermore, we show that it is possible to select for alternative mutations that improve fitness by blocking particularly high-fitness routes to adaptation.

## Introduction

There is a great need for rapid, high-throughput methods to identify adaptive mutations among the growing list of mutations identified in experimentally evolved populations. Several recent ‘Evolve and Resequence’ studies [1], in which populations or clones were sequenced after adaptation to a specific condition, have dramatically increased the list of mutations associated with adaptation to different conditions [2–12]. Within that growing dataset, only a few mutations have actually been confirmed experimentally as adaptive. Some large-scale microbial studies have distinguished adaptive mutations from background neutral mutations on the basis of statistical approaches based on the frequency, enrichment, and recurrence of specific mutations [2, 3, 9, 13–17]. Such statistical approaches entail substantial false-positive and false-negative rates.

Dissecting the fitness effects of every mutation observed in an evolved population is tedious, although generally straightforward. For example, mutations can be reassorted via a genetic cross, and the fitness of segregants carrying individual mutations or combinations thereof can be assessed. That strategy has been used with a few laboratory-evolved *Saccharomyces cerevisiae* clones, demonstrating that evolved clones isolated after several hundred generations of propagation in nutrient-limited conditions often carry one or two adaptive mutations [18, 19]. However, such methods are difficult to scale. An alternative approach is computational models that predict the effects of mutations. A recent study directly compared several popular scoring metrics and found them to be far inferior to experimental testing of fitness [20]. Given its amenability to high-throughput experiments, *S*. *cerevisiae* is particularly well suited for genome-wide assessments of the relationship between genetic variation and fitness. As an alternative, we turned to currently available systematic mutant collections. Researchers have created barcoded strain collections in which thousands of genes are systematically deleted or amplified to uncover gene functions (review in [21]). These strain collections have been used to mimic important classes of mutations such as those resulting in loss-of-function (LOF), gene knockdown, gene duplication, or changes in expression level [22–26]. Missing from these collections are mutations that are not mimicked by copy-number changes, such as mutations in coding regions that generate new protein activities or LOF effects more subtle than those of simple knockout or knockdown alleles. Despite the large number of studies that have used the barcoded collections to detect deleterious effects such as haploinsufficiency, dosage sensitivity, synthetic lethality, drug sensitivity, and various other phenotypes [24, 27–35], only a few studies have looked at beneficial mutations. One study quantified antagonistic pleiotropy in a variety of laboratory conditions and determined that whereas 32% of deletion strains were less fit than a wild-type reference, only 5.1% of the strains were more fit [36]. Another study identified a large number of heterozygous deletions as beneficial but also demonstrated that the haploproficiency was context-dependent [23]. The further application of systematic amplification and deletion collections to study adaptive mutations will expand our understanding of that unique and important class of mutations.

Most previous studies used phenotypic data to investigate gene function. The adaptive phenotypes displayed by the systematic amplification and deletion collections can also be used to investigate questions from an evolutionary genetics perspective. The ability to identify beneficial mutations *en masse* allows us to survey one set of beneficial mutations that could drive adaptation. A greater understanding of adaptive mutations will allow us to begin to address a number of open questions. How does the distribution of fitness effects differ across conditions? What determines which of the possible beneficial mutations actually reach high frequencies in evolving populations? Does the hierarchy of fitness among mutations drive those patterns strictly, or do other factors play a role? How can we better design selective conditions to achieve specific evolutionary outcomes?

We sought to address these questions using a system that combines high-throughput functional genomics and experimental evolution. We first measured the fitness of deletions and amplifications of almost all of the genes in the *S*. *cerevisiae* genome, which we refer to as the amplification and deletion (AD) set, using pooled competitions of thousands of mutants under selection in nutrient-limited continuous culture in chemostats followed by barcode sequencing. We found that while most of the AD mutations were neutral or decreased fitness, ~500 of them increased fitness in at least one condition and hence represented potential adaptive mutations. We next compared the fitness values from the AD set to a set of mutations identified in experimental evolution studies, which we refer to as the evolutionary (E) set. By comparing the E set with the results from the AD set, we recapitulated five of eight previously verified beneficial mutations and predicted that on average at least one third of the mutations present in the evolved strains were likely to positively affect fitness. In sulfate-limited conditions, mutations in one gene dominated the distribution of fitness effects in both the AD set and the E set. In glucose-limited and phosphate-limited conditions, the distributions of fitness effects were characterized by a large number of beneficial mutations of smaller effect. We found that the distribution of fitness effects in the sulfate-limited condition could be modified by precluding the dominant adaptive solution, which allowed the evolving populations to explore alternative beneficial mutations predicted based on the AD set. This study takes an initial step towards determining the fitness effects of candidate adaptive mutations, substantially improving on the throughput of other experimental approaches as well as on the accuracy of purely statistical or computational approaches.

## Results

### A comprehensive survey of single-step mutations (AD set)

**Fig 1:**
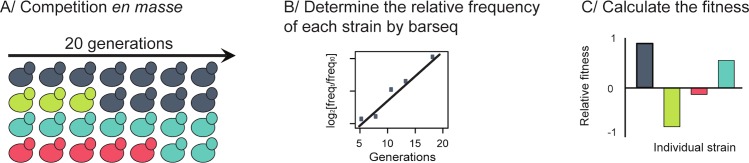
Experimental design for the pooled competition experiments. The proportion of each strain was measured every three to four generations during pooled competition assays, in which all the strains from a single collection were mixed together in equal proportions and grown in continuous culture for 20 generations (**A**). The frequency of each barcode at each time point was measured using the barseq method (**B**). The fitness of each strain was computed based on the measured frequencies (**C**).

We measured the fitness effects of single-gene changes in copy number for ~80% of the genes in the yeast genome using pooled competitions of five different collections of yeast strains in three different nutrient-limited conditions followed by Illumina-based barcode sequencing ([22] **Fig 1**; **S1 Table**). Two of the collections, the deletion collections, consisted of haploid and heterozygous diploid strains, respectively; in each strain, one copy of a single gene was replaced by a selectable marker with a unique DNA barcode [31]. One (control) collection consisted of ~2,000 otherwise isogenic wild-type strains created by placing unique barcodes at a single, neutral genomic location [32]. The other two collections consisted of diploid strains bearing a low or high copy-number plasmid, respectively; each plasmid contained a single gene, the corresponding native promoter, and a unique barcode [29, 30].

We conducted a total of 30 continuous-growth competition experiments with phosphate, glucose, and sulfate, respectively, as the limiting nutrient. We screened each yeast collection twice in each condition (**S1 Fig**). In each screen, we mixed all of the strains from a single collection together at approximately equal proportions in a single culture vessel and measured the proportion of each strain at time points throughout the course of ~20 generations of propagation (**S2 Fig**). We used large populations (~10^9^ cells) to overcome the stochastic effects of drift [23]. We measured the fitness over a relatively short period of time to limit the effects of *de novo* mutations, sampling the populations every three generations to maximize the accuracy of the fitness quantification. We measured the frequency of each strain at each time point using barcode sequencing (barseq; **S3 Fig**) [22]. We note that this experiment design does not allow us to control for mutations already present in the strains before the onset of the competition experiment.

### Hundreds of the copy-number changes in the AD set had positive fitness effects

**Fig 2:**
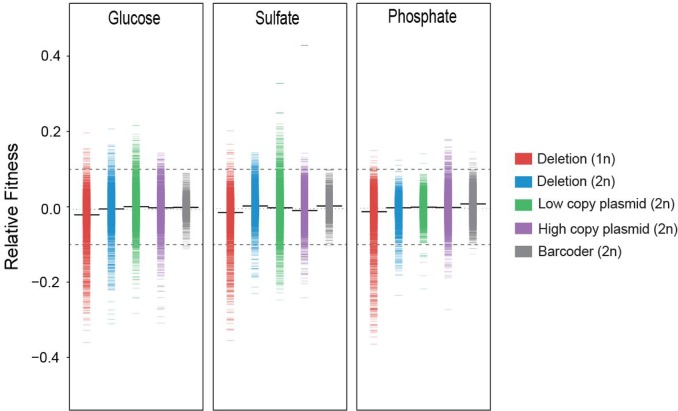
Distribution of the fitness effects of single-gene amplifications and deletions. Fitness distributions of the five yeast collections in glucose-limited, sulfate-limited, and phosphate-limited continuous-growth conditions. The fitness of each strain is shown as a small line. The fitness distribution of the control collection is shown in grey. The thick black line represents the mean. Dashed grey lines indicate the cutoff of ±10% measured using the control collection.

We made a total of 100,853 measurements of relative fitness, ranging from -36.5% to 42.8%, based on an average of 462 reads per gene per screen. We then created fitness distributions of the AD strains in each of the three selective conditions (**Fig 2; Table 1 and S2 Table**). We were able to measure the fitness effects of copy-number changes of 2,133 genes in all 12 experiments and to measure the fitness effects of copy-number changes of an additional 2,953 genes in at least one experiment.

**Table 1:** Number of strains for which fitness was measured in each collection.

Limiting nutrient	Deletion (1N)	Deletion (2N)	Low-copy plasmid	High-copy plasmid
Glucose	3724	5054	4038	4018
Sulfate	3796	5126	4062	3679
Phosphate	4043	5086	4112	4078

To determine the inherent noise originating from the strain construction, pool generation, competition, and sequencing, we quantified the relative fitness of the strains in the control collection. The fitness distribution was tightly centered on 0; 98.2% of the control strains had fitness between -10% and +10% (**Fig 2; S3 Table**). We therefore used fitness values of ±0.10 (corresponding to a 10% change in fitness) as the cutoffs to identify strains in the other four collections that had a significant fitness benefit or deficit compared with the control strains. Previous analyses showed that a beneficial mutation resulting in a 10% fitness increase will reach 5% of the population in ~200 generations and will fix in ~500 generations [37, 38], which suggests that mutations causing a fitness increase of less than 10% would rarely be identified as beneficial in our experimental evolution regime.

Most of the deletion and amplification strains displayed wild-type or near wild-type fitness. The fitness distributions of the AD strains were broader than that of the control strains. Based on the 10% cutoff values, the AD collections were enriched for strains with decreased fitness (n = 1693) or increased fitness (n = 506) compared with the control collection (n = 19 and 80, respectively; Chi square, *p*<0.001 and *p* = 0.0033, respectively; **Fig 2**). Of the strains with increased fitness (**S5 Table**), 223 had increased fitness in sulfate-limited conditions, 210 in glucose-limited conditions, and 73 in phosphate-limited conditions. Only a small fraction of strains had increased fitness in more than one condition (n = 25).

The 506 strains with increased fitness represented copy-number changes in a total of 458 genes (**S5 Table**). Seventy three percent of those strains were from the plasmid collections, which comprised just 47% of the total strains tested, suggesting that duplications of single genes are more likely than deletions to produce fitness gains. The AD set only recreates gross dosage changes and not mutations acting via different mechanisms; however, our screen identified five of eight genes in which beneficial mutations were previously identified in evolution experiments (considering only those known beneficial mutations with matching strains in the AD set): the amplification of *SUL1* and LOF mutations affecting *SGF73* in sulfate-limited conditions and mutations affecting *MTH1*, *WHI2*, and *GPB2* in glucose-limited conditions [18, 19, 39]. These results demonstrate that the AD collections were able to replicate the phenotypes caused by some beneficial mutations, although they failed to replicate those caused by others (e.g., mutations in *PHO84*, *IRA1*, and *RIM15*). Among the genes associated with a fitness increase in the AD set, *SUL1* was associated with the greatest fitness (42.8% in the sulfate-limited condition for a strain carrying the high-copy plasmid). In previous experiments, *SUL1* amplification was recurrently selected during evolution in sulfate-limited conditions, and increasing the *SUL1* copy number via expression on both low-copy and high-copy plasmids increased fitness [39, 40]. Our screen also identified one gene that was previously identified as the cause of putative secondary adaptive effects: *BSD2*, a gene involved in the downregulation of the metal transporter proteins Smf1 and Smf2 [41, 42] and located 6kb upstream of *SUL1* on chromosome 2. The amplification of *BSD2* on a low-copy plasmid increased fitness by 5% and 12.4% in the sulfate-limited and glucose-limited conditions, respectively. In previous studies of the *SUL1* amplicon [39, 40], we detected only three independent clones where the *SUL1* amplicon excluded *BSD2*. The fitness of each of 13 strains harboring an amplification of both *SUL1* and *BSD2* was higher than the fitness of three strains harboring an amplification of *SUL1* but not of *BSD2* [40], a result that was further supported by a fitness analysis of synthetic amplicons [19]. The reintroduction of *BSD2* using a low-copy plasmid into one of the three strains harboring only *SUL1* amplification increased the fitness in the sulfate-limited condition by 6.1% (from 37.7% to 43.8%), suggesting that the fitness effects of the two mutations are additive. These results demonstrate that the AD screen is able to detect adaptive mutations even of small effect, although our control experiments suggest that the identification of such mutations is likely subject to a higher false-positive rate than the identification of beneficial mutations of larger effect. A decrease in the cutoff to ±5% resulted in the identification of increased or decreased fitness in 15% of the control strains and increased the number of beneficial mutations identified in the AD collections by six fold (n = 3143). Although the less stringent cutoff still identified significantly more beneficial mutations in the AD collections than in the control collection (Chi square, *p*<0.001), we decided to use the more stringent cutoff to focus on the mutations with the highest impact.

Next, we sought to apply the knowledge gained from the screen of the AD set to the hundreds of *de novo* mutations identified in laboratory evolution experiments (E set). Our goal was to determine which of the hundreds of possible adaptive mutations identified in the AD set were actually selected during experimental evolution.

### Mutations identified in evolution experiments (E set)

To compare the genes in the AD set that we identified as potential sites of adaptive mutations to the genes in which mutations actually occurred during experimental evolution, we first needed to create a comprehensive database of mutations identified in yeast evolution experiments. To do so, we identified and resequenced the mutations that occurred in yeast evolution experiments carried out by our lab [39, 40]. The experiments involved the propagation of haploid or diploid prototrophic strains of *S*. *cerevisiae* for 122 to 328 generations in continuous-culture conditions identical to those in which our AD screens were performed (six sulfate-limited, six phosphate-limited, and four glucose-limited populations. We detected 150 mutations by whole-genome sequencing of 16 populations and 34 clones (See **Materials and Methods**). We then collected a large set of mutations from various Evolve and Resequence studies of yeast performed in a variety of conditions [2–4, 8, 40, 43]. Thus, we compiled a total of 1,167 mutations in 1,088 genes from 106 long-term evolution experiments conducted in 11 different conditions in nine previous studies. We refer to this set of mutations as the E set (**S4 Table**). The features of the previous studies and the resulting mutations are summarized in **Table 2**. The complete list of mutations, their frequencies, and their predicted effects are given in **S4 Table**. The E set did not include chromosomal rearrangements, because those events were not always reported in the previous studies.

**Table 2:** Mutational catalog (E set) subdivided by conditions, ploidy, and sample type.

		Number of mutations compiled	From this study
**Conditions**	YPD	720	NA
	Glucose	224	23
	Sulfate	97	76
	Phosphate	54	51
	Other	72	NA
**Ploidy**	Haploid	1017	75
	Diploid	150	75
**Sample type**	Clones	305	75
	Population	862	75

### Loss-of-function mutations were enriched in haploids and were depleted and recessive in diploids

Two recent studies showed that LOF mutations were frequently selected in populations of haploid yeast [2, 3]. Based on a small number of mutations, another study concluded that mutations affecting cis-regulatory regions are co-dominant in heterozygous diploids [44]. Although those results are suggestive, too few Evolve and Resequence studies have been performed in diploid yeast to draw firm conclusions about the effects of ploidy on the distribution of fitness effects.

We divided the E set into four groups based on SNPeff, an annotation program that predicts the functional impact of the mutation of a gene, as follows [45]: (1) high-impact mutations, such as frameshifts or the gain or loss of a start or stop codon; (2) moderate-impact mutations, such as non-synonymous substitutions or the deletion or insertion of a codon; (3) low-impact synonymous mutations; and (4) modifiers, corresponding to mutations upstream of a gene or within intergenic regions. We found that different types of mutations tended to be present in haploid and diploid strains, respectively (Fisher’s exact test, *p*<0.001, corrected for multiple tests). We confirmed previous findings showing that in haploids, the main category of mutation is LOF mutations involving the gain of a stop codon (Chi square, *p* = 0.003; **Table 3**). In contrast, LOF mutations were relatively rare in diploid strains, which were instead enriched for intergenic and upstream mutations (Chi square, *p*<0.001; **Table 3**), suggesting that amplifications and gain-of-function (GOF) mutations are more important in the diploid background. This result is consistent with our previous observations that evolved diploid strains contain more and larger variations in gene and chromosome copy numbers than evolved haploid strains [39]. Using only the mutations identified in glucose-limited conditions from the E set, we determined that the mutational signature was different between haploids and diploids in glucose-limited conditions (Fisher’s exact test, *p*<0.001), with an enrichment of LOF mutations among the haploids (Chi-square, n = 224, *p*<0.001). Conversely, the mutations identified in phosphate-limited conditions in the E set displayed only marginal enrichment of LOF mutations (Fisher exact test, n = 54 *p* = 0.053), while those identified in sulfate-limited conditions displayed no enrichment of LOF mutations (n = 100).

**Table 3:** Comparison of the mutational signature between haploid and diploid strains (E set).

Class	All	Diploid	Haploid	*p*-value*	q-values*	SNPeff
Stop gained	123	5	**118**	0.003	0.005	High
Start lost	8	1	7	0.97	0.591	High
Stop lost	2	0	2	0.59	0.469	High
Frameshift	6	0	6	0.74	0.496	High
Non-synonymous substitution	817	97	720	0.15	0.201	Moderate
Codon deletion/insertion	3	0	3	0.51	0.469	Moderate
Synonymous substitution	142	15	127	0.46	0.469	Low
Upstream	13	**7**	6	0.0001	0.002	Modifier
Intron	5	1	4	0.63	0.469	Modifier
Intergenic	48	24	24	0.0001	0.002	Modifier
Total	1167	150	1017	<0.001	<0.001	

* Chi-square and ¥ Fisher's exact test *p*-values

The different types of mutations observed between ploidies are likely explained by the tendency of LOF mutations to be recessive [46, 47] compared with mutations that increase gene expression, which are more likely to have an effect in heterozygotes. Although loss of heterozygosity has been observed in diploid populations [39, 46], such cases are relatively rare. To test that directly, we examined the fitness effects of 55 beneficial deletions identified in both the haploid and the diploid AD collections and found that those deletions indeed tended to be recessive, causing on average a 9.0% ± 4.6 greater fitness increase in haploids than in diploids. Seven of the 55 deletions (*WSC3*, *TIM12*, *IPT1*, *MMS22*, *NDL1*, *PBS2*, and *YLR280C*) had the same fitness effect in haploids and diploids, indicating that a subset of LOF mutations can in fact be dominant. Overall, LOF mutations appeared to provide a greater adaptive benefit in haploid strains than in diploid strains, which is consistent with prior results.

### Mutated pathways are constrained

**Fig 3:**
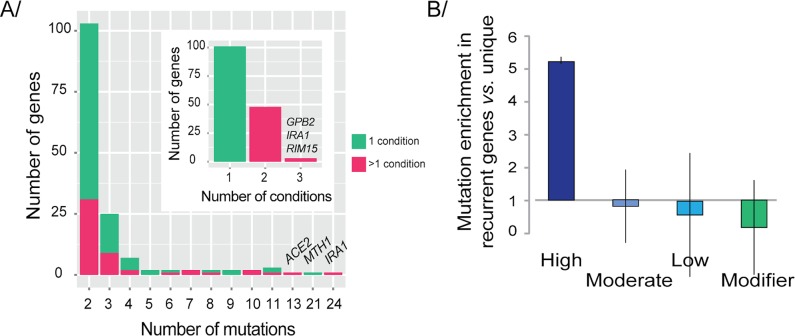
Recurrently mutated genes reveal how evolution is constrained. (**A**) Repeatability of adaptation and parallelism at the gene level. Genes were classified by the number of mutations detected during Evolve and Resequence studies: 154 genes were mutated in more than one sample; 48 genes with recurrent mutations were mutated in more than one condition (small panel). (**B**) Enrichment of recurrently mutated genes with high-impact mutations compared with genes mutated in only one sample. Enrichment is not observed for moderate or low impact mutations, or modifiers. Error bars are 95% CIs.

Recurrence-based models, which assume that oncogenes are recurrently mutated among independent samples, are one of the most widely used approaches to identify putative driver genes in cancer [48–50]. Recurrent adaptive trajectories have also been frequently observed in microbial evolution [2, 3], leading to the discovery of drivers of adaptation such as *SUL1*, *HXT6/7*, and *RIM15* in *S*. *cerevisiae* and *rpoS* in *Escherichia coli* [3, 13, 14, 39, 51]. Of the 1,088 genes in the E set, 154 were mutated in more than one sample, and 19 were mutated in more than five samples (**Fig 3A, S4 Table).** The recurrently mutated genes were highly enriched with high-impact mutations (Fisher’s exact test, *p*<0.001; **Fig 3B**) and tended to be longer than genes that were mutated in only one sample (Wilcoxon rank-sum test, *p*<0.001; **S4A Fig)**. There are several tools that correct for gene length to detect true adaptive mutations and discard false-positives [52]. We decided to use a different approach by inferring the fitness effects of mutations using the results from the AD screen.

### Prediction of evolutionary responses to strong selection

Convergent evolution has been widely used as a predictor of evolutionary outcomes. We decided to compare the list of recurrently mutated genes from the E set to the results of the AD screen, restricting our analysis to experiments performed in the same conditions.

In the E set, 36 genes were mutated twice in at least one of the three conditions used in the AD screen. Ten of those genes were associated with a fitness increase of at least 10% in at least one collection in the AD set (*SUL1* and *SGF73* in the sulfate-limited condition and *GPB2*, *PBS2*, *AEP3*, *MUK1*, *HOG1*, *ERG5*, *SSK2*, and *WHI2* in the glucose-limited condition). Eight more genes were associated with a fitness increase that did not meet our stringent cutoff of 10% but exceeded 5%. The remaining 18 genes were either absent from the collections (n = 12) or associated with no fitness increase in the corresponding condition (n = 6). The six genes that showed no fitness effect in the AD set could have been recurrently mutated by chance. Alternatively, the mutations in the E set could have provided fitness increases that were not mimicked by the AD collections, which could be the case for mutations that caused partial LOF or that resulted in a novel function, or due to fitness-changing errors or secondary mutations in the relevant strains. Another possibility is that those mutations only provided a benefit in a specific genetic background or in concert with other mutations. Strains from the AD set could also have accumulated additional mutations that mask the true effect of the query mutation.

A large number of genes identified in the E set were mutated only in a single population. Because the number of Evolve and Resequence experiments is relatively small, akin to a non-saturating genetic screen, some adaptive mutations are likely to be found as singletons and would therefore be missed by a recurrence-based detection method. The E set contained 155 genes that were mutated only once in glucose-limited, sulfate-limited, or phosphate-limited conditions. We used the data from the AD set to determine if those singletons might be associated with a fitness increase in the corresponding environment. Of the 155 singletons, only three had a fitness effect of at least 10% when amplified or deleted: amplifications of *NMA111* in the sulfate-limited condition and *CLN2* and *YOR152C* in the glucose-limited condition. Thirty-eight more genes had a fitness effect between 10% and 5% (average fitness = 7.2±1.1). Cln2 is one of the three G1 cyclins and promotes cell-cycle progression. The expression of G1 cyclins is regulated in response to nutrient limitations; in particular, it is repressed in the presence of glucose [53].

These results show that while convergent evolution is useful for identifying adaptive mutations, some singletons might also have fitness effects and should not be overlooked. Only a small portion of the singleton mutations were predicted by the AD screen to be beneficial, suggesting three possibilities, which are not mutually exclusive: the relevant data are missing from the AD screen (only 52 of the 202 genes with singleton mutations were represented in all four collections and all three conditions used for the AD screen); the AD screen does not accurately reflect the fitness of these point mutations; or the singletons were increasing in frequency in the evolved populations due to the presence of a beneficial mutation elsewhere in the genome, a phenomenon known as hitchhiking. If the first or second explanation were true, many of the evolved samples should lack mutations predicted to be adaptive by the AD screen, because the AD screen would have a high false-negative rate. If most of the singletons were the result of hitchhiking, all of the evolved samples should carry mutations predicted to be beneficial by the AD screen in addition to the neutral or weakly deleterious hitchhiker mutations.

### All evolved populations harbor beneficial mutations

**Fig 4:**
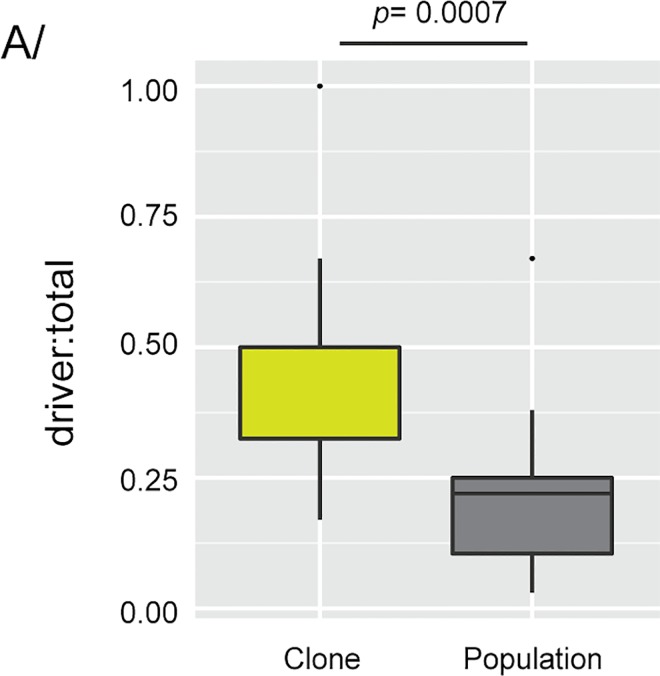
Driver mutations. (**A**) Boxplot representing the ratio of driver to total mutations detected in evolved clones and populations. The significance of the difference between clones and populations was estimated using a Wilcoxon-ranked test.

In order to determine the relative contributions of these explanations, we predicted the number of adaptive mutations each population and clone in the E set should carry based on the frequency of recurrence in the E set and the fitness data from the AD set. We determined that each clone or population in the E set carried at least one adaptive mutation predicted by the AD screen, which is consistent with the modest false-negative rate for the AD screen. Each sample in the E set contained on average 1.8 (2.2 per population and 1.4 per clone) adaptive mutations predicted by the AD screen, representing 35% of the total mutations identified in the E set (**Fig 4A–S6 Table**). There was no difference in the prevalence of predicted adaptive mutations among the three selective conditions (**S4B Fig**). That result is consistent with previous reports of frequent hitchhiking by neutral or deleterious mutations [2, 51, 54, 55]. Our estimate largely agrees with the results of detailed genetic analyses of mutations carried by evolved strains, which found that one third of the single-gene mutations among a total of five evolved clones were associated with a fitness increase [18, 19, 39]. Thus, by combining the data from the AD set and the E set, we were able to generate a more comprehensive list of adaptive mutations in evolved populations as well as estimate the genomic reservoir of beneficial mutations that were not detected. We conclude that evolution is partly predictable based on the repeatability of adaptive mutations among independent populations and reflects, at least in part, the fitness distribution of possible mutations, as mimicked by genome-wide screens of gene deletions and amplifications.

### The set of beneficial mutations reveals potential drivers of adaptation

The E set defined a set of 28 genes that were the sites of adaptive mutations with large effects (based on the classification of mutations present in the AD set), which we consider to be candidate driver genes. Three of the candidate driver genes were mutated in only one sample, and 25 were mutated repeatedly among different samples. The AD screen identified a large number of potential sites of beneficial mutations that were a single mutational step away from the ancestral genotype [56]. To determine what differentiates the actual mutational spectrum from the pool of potential beneficial mutations, we excluded the genes in the E set that harbored mutations that were predicted to be beneficial based on the AD screen (n = 28) and examined the remaining genes that were associated with fitness increases in the AD screen (n = 430). Given the population sizes (10^5^ to 10^10^ cells) and numbers of generations (50 to 1000) in the evolution experiments and the size of the yeast genome (~12 megabases), it is likely that every base in the genome was mutated at least once at some point among the ensemble of experiments in the E set. It therefore seems unlikely that mutations in the 430 genes identified in the AD screen as potential sites of adaptive mutations failed to occur at some point in the evolution experiments, although there was a greater likelihood that mutations mimicking the plasmid-based amplifications actually failed to occur, because point mutations that significantly increase gene expression might simply not exist in some promoter regions [57]. Furthermore, gene-amplification rates are generally biased by genomic-architecture constraints, such as proximity to repeat sequences, and the fitness effects of multigenic amplicons are complicated by the contributions of genes linked to the driver gene [19].

**Fig 5:**
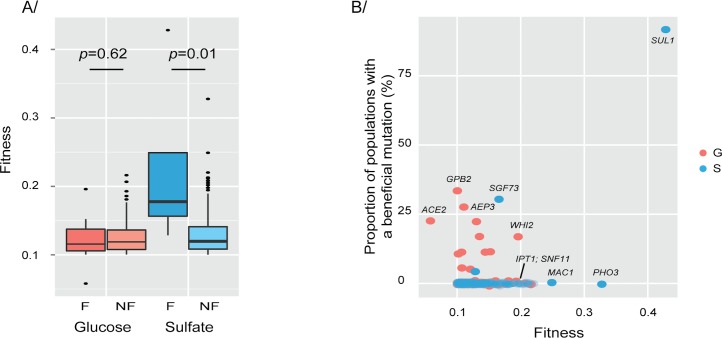
Alternative accessible evolutionary paths. **(A)** The fitness of beneficial mutations found (F) in Evolve and Resequence studies is significantly higher than the fitness of beneficial mutations not found (NF) in sulfate-limitation but not in glucose-limitation. The significance of the difference between the two boxplots for each condition was estimated using a Wilcoxon-ranked test. (**B)** Each point represents the fitness of a strain and the proportion of Evolve and Resequence samples with the corresponding gene mutated. *SUL1* dominates the fitness and mutational spectrum. Several mutations have a high fitness but have never been detected in Evolve and Resequence studies and might correspond to potential drivers of adaptation.

In order to better understand those issues, we compared the condition-specific fitness effects of the AD mutations that matched E-set mutations in the same condition with those of the AD mutations that did not match any E-set mutations in the same condition. In the glucose-limited condition, there was no difference on average between the fitness effects of the AD mutations with and without matching E-set mutations (**Fig 5A)**. In the sulfate-limited condition, the AD mutations with matching E-set mutations had greater fitness effects on average than those without matching E-set mutations (Wilcoxon rank-sum test, *p* = 0.001; **Fig 5A**). Consistent with previous findings, *SUL1* dominated the fitness distributions in sulfate-limited conditions in both the AD set and the E set (**Fig 5B**). When the *SUL1* amplifications were excluded from the comparison of AD mutations with and without matching E-set mutations, the AD mutations with matching E-set mutations still had greater fitness effects on average than those without matching E-set mutations (Wilcoxon rank-sum test, *p* = 0.05). Other highly beneficial mutations (with >20% fitness increase) such as amplifications of *MAC1* and *PHO3*; encoding proteins implicated in copper and phosphate-sulfate metabolism, respectively; appear to be potential drivers of evolution but have not been identified in evolved populations (**Fig 5B;** [2, 58]). That suggests that, at least under sulfate-limited conditions, adaptation can be predicted based on the fitness effects of potential single-gene mutations, with the mutations providing the largest increase in fitness being the most likely to reach high frequencies. Although fewer clones and populations have been sequenced from phosphate-limited evolution experiments, all of the beneficial mutations in that condition in the E set could be predicted based on recurrence. Conversely, in glucose limitation, a variety of beneficial mutations with smaller fitness effects appear to be possible and were indeed observed in evolved populations.

Condition-dependent or genome-wide variation in mutation rates could bias adaptive outcomes relative to the distribution of fitness effects seen in the AD screen [2]. The lack of observed mutations in the E set corresponding to many of the genes identified by the AD screen as potential sites of beneficial mutations likely reflects a combination of many factors, including random chance, epistatic interactions, strain background differences, or a failure of the AD set to adequately recapitulate the fitness of *de novo* mutations. Clonal interference is also likely to play a role.

### Mutational spectrum in the absence of the main adaptive mutation

**Fig 6:**
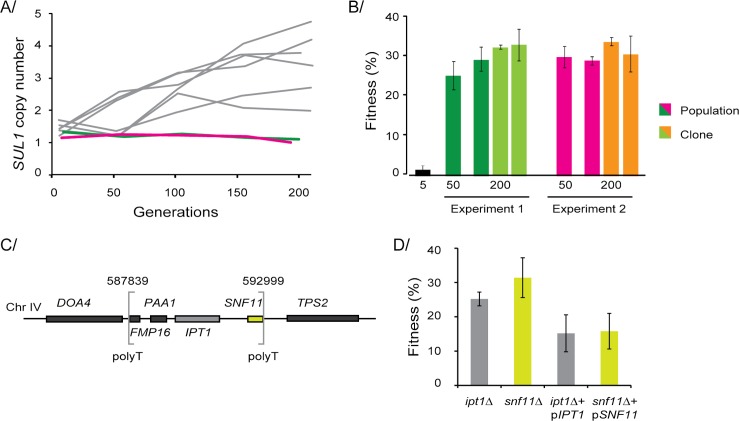
Alternative beneficial mutations are selected in the absence of the main driver. (**A**) The copy number of *SUL1* was assessed using qPCR of samples taken from two independent experiments in which *SUL1* was not amplified (green and pink) and compared with previously published data from wild-type strains (in grey) [40]. (**B**) The fitness coefficient as compared to the ancestral strain of population samples at generations 5, 50, and 200 and the fitness of two clones isolated at generation 200. (**C**) A small deletion (~5kb) encompassing four genes on chromosome IV was detected in a population from one experiment (between brackets); polyT sequences are present at the breakpoints. The colors of the boxes represent the orientation of the genes (yellow: gene on the Watson strand, grey: genes on the Crick strand). (**D**) Fitness coefficients of the deletion strains *ipt1*Δ and *snf11*Δ and those of both deletion strains complemented with *IPT1* or *SNF11* on a low-copy plasmid grown in sulfate limitation.

We asked which mutations would be selected in sulfate-limited conditions if *SUL1* amplification were not possible. Alternative adaptive mutations might only rarely reach high frequencies in sulfate-limited conditions because of the strong fitness effects of *SUL1* amplification. We hypothesized that in the absence of the *SUL1* amplification, a variety of alternative mutations of smaller effect would be selected, an outcome more similar to the pattern observed in glucose limitation. We analyzed two populations that lacked *SUL1* amplifications (**Fig 6A,** population s611 and s612 **S4 Table**) but showed fitness gains after 200 generations of evolution in sulfate-limited conditions. The fitness gains of those populations (~30%; **Fig 6B**) were near the lower end of the range of fitness gains in previously studied clones harboring *SUL1* amplifications (37–53%) [40]. To establish which mutations were responsible for the fitness gains in the absence of *SUL1* amplification, we performed whole-genome sequencing of the populations isolated at generation 200. We detected two independent, non-synonymous mutations (N263H and N250K) in the coding region of *SUL1* in both populations (**S4 Table**). We inserted each of those mutations into wild-type strains and found that N250K increased fitness by 23.1% (±2.3%) and N263H increased fitness by 17.7% (±1.22%). In addition, one population (s611) harbored a nonsense mutation in *SGF73*, a gene previously identified as the site of an adaptive mutation (**S4 Table**), and the other population (s612), harbored a 5.1 kb deletion on chromosome IV (587839–592999) affecting four genes (*FMP16*, *PAA1*, *IPT1*, and *SNF11*; **Fig 6C**). In the AD screen, deletions of *IPT1* and *SNF11* were beneficial in glucose-limited and sulfate-limited conditions (10–20% fitness increase), but mutations in those genes were not included in the E set (**Fig 5B**). Because *IPT1* and *SNF11* are adjacent to one another on the chromosome, we suspected that one of them might be a false positive, resulting from a known artifact called the neighboring gene effect [59]. By employing complementation testing using centromeric plasmids, we found that the deletion of either gene increased fitness (**Fig 6D**). Snf11 is a subunit of the SWI/SNF chromatin remodeling complex, which is known to act as a tumor suppressor in humans [60]. Ipt1 is implicated in membrane-phospholipid metabolism and nutrient uptake [61]. Thus, our results showed that adaptive mutations predicted by the AD screen can be relevant, even when they are rarely identified in evolution experiments. We predict that additional evolution experiments that preclude the possibility of *SUL1* amplification will reveal even more alternative fitness peaks.

## Discussion

We addressed two central topics in evolutionary biology: the relationship between genotype and fitness and evolutionary constraints despite the presence of alternative evolutionary paths.

### The high-throughput functional screen improved the detection of adaptive mutations

The recurrence-based identification of adaptive mutations provides an incomplete picture of the impact of mutations on cellular fitness [62]. In agreement with previous reports [2, 3, 9, 13, 39, 51], we found that experimental evolution resulted in non-uniform selection of mutations across the genome (**Fig 3A**). It is currently impossible to screen all possible mutations, so we used whole-gene amplifications and deletions as a first step in approximating the spectrum of potential mutations. We believe that this is a reasonable approach given the prevalence of gene copy-number changes and LOF mutations in experimentally evolved populations [2, 3, 39], and our success in identifying genes with previously validated high fitness mutations.

Our results can be used to prioritize the experimental validation of potentially adaptive mutations found in evolved strains. The AD screen allowed us to discriminate between adaptive mutations and neutral or passenger mutations in evolved populations. Based on the results of the AD screen combined with the information provided by the E set, we predict that ~35% of the mutations appearing in laboratory-evolved populations are likely beneficial. As expected, that number is higher than previous estimates of the baseline rate of beneficial mutations (6–13%) based on mutation-accumulation experiments with yeast [63].

### Different functional categories of mutations are selected based on ploidy

The frequencies of different categories of adaptive mutations (e.g., LOF or altered level of expression) differed between haploids and diploids. In agreement with previous work [3], we detected an excess of LOF mutations in haploids and an excess of mutations that likely modify gene expression in diploids. Our results agree with those of several studies showing that mutations have greater fitness effects in haploids than in heterozygous diploids [64] and that the frequency of fixation is higher in diploids [37]. Mutations affecting cis-regulatory regions have often been described as co-dominant, whereas most mutations in coding regions cause LOF and are recessive [44]. Large copy-number variations (CNVs) have been shown to be enriched in diploid backgrounds compared with haploid backgrounds [39], suggesting that a diploid context might buffer the detrimental effects of aneuploidy and CNVs seen in haploids [65, 66]. These results emphasize the point that evolutionary trajectories are constrained by ploidy and that patterns observed at a particular ploidy are unlikely to act universally.

We also observed that the majority of the beneficial mutations from the AD set are from the plasmid collection, further illustrating the importance of gene amplifications in adaptation.

### Remaining open questions

Despite our promising results, functional screens using single-gene amplifications and deletions have several limitations. The available yeast collections are based on single-gene copy-number changes and do not allow the study of mutations in protein-coding regions that are not mimicked by dosage changes, mutations in non-genic functional elements, or combinations of mutations. To explore the importance of non-genic regions and small genes that are not present in the yeast collections, billions of individual and combined mutations need to be generated in a comprehensive way, similar to the deep mutational scanning of proteins [67], the Million Mutation Project [68], or newly created resources such as the tRNA deletion collection [69] and large telomeric amplicons [19]. Previous studies in microbial and viral systems have provided evidence for both antagonistic and synergistic epistasis among beneficial mutations [36, 70–73]. Synthetic genetic arrays and similar approaches using the *S*. *cerevisiae* deletion collection have been used to characterize negative and positive epistatic relationships, and a nearly complete yeast genetic-interaction network has been generated using double mutants [74, 75]. Further studies using those resources will allow us to move beyond single-gene effects and begin to understand how interactions among multiple genes in CNVs and combinations of mutations shape the distribution of fitness effects. By expanding and developing these techniques, the increase of studies combining long-term experimental evolution and whole-genome sequencing will likely reveal additional mutational effects.

## Materials and Methods

### Strains and media

The MoBY-ORF collection of centromeric (CEN) plasmids in *E*. *coli* was obtained from Open Biosystems and stored at -80°C as individual strains in 96-well plates. The plates were thawed and robotically replicated onto LB-Lennox (Tryptone 10g, yeast extract 5g, NaCl 5g) agar plates containing 5Δg/ml tetracycline, 12.5μg/ml chloramphenicol, and 100μg/ml kanamycin and grown at 37°C for 14 h. Colonies were harvested by addition of 5ml LB-Lennox to each plate and subsequently pooled. Glycerol (50%) was added, and 1ml aliquots containing 2×10^9^ cells were frozen at -80°C. Plasmid DNA was prepared from the *E*. *coli* pool and then used to transform the *S*. *cerevisiae* S288C derivative strain DBY10150 (*ura3-52*/*ura3-52*) using a standard lithium acetate protocol. The yeast cells were selected on -URA and 200μg/ml G418 plates, resulting in 88,756 transformants, which were then pooled together, giving an average library coverage of **~**20×. The MOBY-ORF v2.0 collection (2 micron plasmid) was obtained from the Boone lab and crossed for 3 h with YMD1797 (*MATα*, *leu2Δ1*). Clones were selected twice on MSG/B and G418 (200μg/ml) and then pooled together. The *MATa/MATα* SGA Marker (MM2N) collection was obtained already pooled from the Spencer lab. The *MATa* SGA Marker (MM1N) library was obtained frozen from the Caudy lab; the strains were selected on -LYS and -MET and then pooled together. The barcoder collection was obtained frozen from the Nislow lab. The plates were thawed at room temperature, replicated onto YPD and G418 (200μg/ml), and crossed with FY5 (*MATα*, prototrophic strain). The strains were then selected twice on MSG/B+G418 (200μg/ml) and pooled together. A list of the strains used in this study can be found in **S1 Table**.

### Continuous cultures and pooled competition experiments

Previously described nutrient-limited media (sulfate-limited, glucose-limited, and phosphate-limited [13, 39, 76]) were complemented with uracil and histidine (20mg/l) for the SGA Marker pools. For each competition, a 200ml culture was inoculated with 1ml of a single pool (~2×10^7^ cells). Two competition experiments were performed for each pool. The cultures were grown in chemostat culture at 30°C with a dilution rate of 0.17±0.01 volumes/h. The cultures were grown in batch for 30h and then switched to continuous culture. The continuous cultures reached steady state after ~10 generations and were maintained for an additional 20 generations (**S2 Fig**). A sample taken just after the switch to continuous culture was designated generation 0 (G0). Subsequent samples were harvested every three generations thereafter. Samples for cell counts and DNA extraction were passively collected twice daily.

### Genomic DNA preparation, plasmid extraction, and qPCR

Genomic DNA was extracted from dry, frozen cell pellets using the Smash-and-Grab method [77]. Plasmids from the MoBY collections were extracted with a Qiagen miniprep protocol (QIAprep Spin mini prep kit; Qiagen, Hilden, Germany) with the following modification: 0.350mg of glass beads were added to a cell pellet with 250μl buffer P1 and vortexed for 5min. Then, 250μl buffer P2 was added to the mix of cells and beads, and 350μl buffer N3 was added to the solution before centrifuging for 10 min. The supernatant was then applied to the Qiagen column following the recommendation of the Qiagen miniprep kit. Plasmid DNA was then eluted in 50μl sterile water. Genomic DNA was extracted from dry cell pellets by the Smash-and-Grab method and used for barcode verification of single strains by PCR amplification and Sanger sequencing as previously described [40]. For each sample, the plasmid copy number was determined using the copy number of *KanMX* relative to the copy number of *DNF2*, a gene located on chromosome 4 and absent from the two MoBY collections (see **S6 Fig**). The primers used are listed in **S8 Table**. Microarray assays, whole-genome sequencing, SNP calling, and qPCR analysis were performed as previously described [40]. The microarray data have been deposited in the Gene Expression Omnibus repository under accession GSE58497 (http://www.ncbi.nlm.nih.gov/geo/query/acc.cgi?token=sjgtsgwmdhajdud&acc=GSE58497). The fastq file for each library is available from the NCBI Short Read Archive with the accession number PRJNA248591 and BioProject accession PRJNA249086.

### Barseq experiments and fitness measurement

Amplifications of the barcodes were performed using a modified protocol [22]. Uptag barcodes were amplified using primers containing the sequence of the common barcode primers (bold), a 6-mer tag for Illumina multiplexing (in italics), and the sequence required for attachment to the Illumina flowcell (underlined; **S8 Table)**. PCR amplifications were performed in 100μl, using Roche FastStart DNA polymerase with the following conditions: 94°C for 3min; 25 cycles of 94°C for 30s, 55°C for 30s, and 72°C for 30s; followed by 72°C for 3min. PCR products were then purified using the Qiagen MinElute PCR Purification kit (cat. No. 28004), quantified using a Qubit fluorometer, and then adjusted to a concentration of 10μg/ml. Equal volumes of normalized DNA were then pooled and gel purified from 6% polyacrylamide TBE gels (Invitrogen) using a soak and crush method followed by purification and concentration using Qiagen Qiaquick PCR purification. After quantification using a Qubit fluorimeter, the libraries were sequenced using the standard Illumina protocol as multiplexed, single-read, 36-base cycles on several lanes of an Illumina Genome Analyser IIx (GAII). Thirty multiplexed libraries (UPTAGS only) were sequenced on several lanes of an Illumina GAII. An average of 25,664,072 million reads per library that perfectly matched the molecular barcodes were obtained (**S9 Table)**. The fastq file for each library is available from the NCBI Short Read Archive with the accession number PRJNA248591 and BioProject accession PRJNA249086 (**S10 Table)**. The 6-mer multiplexing tags were reassigned to a particular sample using a custom Perl script (**S1 File**). Then, each barcode was reassigned to a gene using a standard binary search program (programmed in C, **S2 File**). Only reads that matched perfectly to the reannotated yeast deletion collection [22] or the MoBY-ORF collection [29] were used. For the barcoder collection, 1885 barcodes were recovered using a compiled list of all barcodes previously published (1624 barcodes from the barcode list of the deletion collection and 260 barcodes from the Yeast Barcoders collection; [28, 32]). Multiple genes with the same barcodes were discarded. Strains with less than 20 counts across the different samples were discarded. The numbers of strains identified for the five collections in the three conditions are summarized in **S9 Table**. To avoid division by zero errors, each barcode count was increased by 10 before being normalized to the total number of reads for each sample. To quantify the relative fitness of each strain during growth in the various conditions, the analysis was restricted to the time during which the populations were in a steady-state phase, defined as generations 6 through 20. Generation 0 was used as t_0._ The linear regression of the log_2_ ratios of the normalized barcode counts at generations 6–20 to that at generation 0 was used to calculate the fitness of each strain. The two replicate measurements were then averaged. The source code is provided in the Supporting Information (R script, **S3 File**). The correlation between each pair of replicates was displayed using the R package corrgram. The distribution of the averaged fitness was displayed using the R package beanplot [78].

### Validation of the fitness measurements and pairwise competitions

To ensure that the pooled fitness measurements accurately reflected the fitness of each strain, the relative fitness of 51 strains from the deletion and plasmid collections that had deleterious, neutral, or beneficial changes was measured by pairwise competitions against a control strain marked with a fluorescent protein (eGFP) in the three conditions used in the pooled experiments. Fitness measurements of the individual clones were performed as previously described [40] using FY strains in which the *HO* locus was replaced with *eGFP* (*MATa*: YMD1214 and *MATa*/*MATα*: YMD2196; **S5 Fig, S7 Table**). The fitness values were similar in both assays, and there was a strong positive correlation (R^2^ = 0.83) between the fitness values from the large pool screen and the pairwise fitness assays (**S5 Fig** and **S7 Table**). To limit artifacts due to preexisting mutations or copy-number changes in the genomes of the pooled strains, most of the barcoded pools were created either by fresh transformation (in the case of the plasmid collections) or from a fresh cross of the commercially available collection stocks with a wild-type strain (see the **Materials and Methods**).

To detect the extent of extraneous mutations in the validation panel, 51 strains were screened for the most common secondary mutation detected previously in the deletion collection: mutations in *WHI2*, which is involved in the regulation of cell proliferation [79]. Mutations in *WHI2* were screened in the 51 strains by PCR using oligo (YOR043W-for and YPR043W-rev) and Sanger sequencing (**S7 Table**). Microarray analysis of the last sample of one of the competitions of the low-copy plasmid collection was used to verify that there were no copy-number changes, other than those due to the plasmids, at the population level; although that approach would only detect CNVs that achieved at least a ~10% frequency in the population.

### Data access

All sequencing data from this study have been submitted to the NCBI Sequence Read Archive (SRA; http://www.ncbi.nlm.nih.gov/sra) under accession number PRJNA248591 and BioProject accession PRJNA249086. Microarray data from this article have been deposited in the Gene Expression Omnibus repository under accession GSE58497 (http://www.ncbi.nlm.nih.gov/geo/query/acc.cgi?token=sjgtsgwmdhajdud&acc=GSE58497).

## Supporting Information

S1 Fig Scatter plots of fitness between replicates for each condition and pool. Each experiment is labeled with the condition (G, S, or P, for glucose, sulfate, or phosphate limitation) and the replicate (1 or 2).
(PDF)

S2 Fig Steady state in continuous cultures was reached at generation six. Cell density over time is shown for each pool grown in glucose, sulfate, and phosphate limitation for 20 generations.
(PDF)

S3 Fig Relative frequency over time of three strains from four collections. Each box, represents the relative frequency (log_2_ ratio of the frequency) of one strain over time. Each line (blue and red) represents the linear regression used to calculate the relative fitness between generations 6 and 20.
(PDF)

S4 Fig Distribution of high-impact mutations. (**A**) Distribution of gene size for recurrently mutated genes and genes mutated in only sample, respectively. The significance of the difference between the two boxplots was estimated using a Wilcoxon rank-sum test. (**B**) The ratio of driver mutations to total mutations was not condition-specific (*p* = 0.61, 0.05, and 0.05 for glucose limitation, sulfate limitation, and phosphate limitation, respectively).
(PDF)

S5 Fig Fitness of 51 mutant strains measured in pooled competitions by barseq and in pairwise competition assays. The fitness values in the pooled experiments are relative to the mean fitness of the population. We therefore compared the fitness of 51 strains measured in the pooled assays to that measured in pairwise fitness assays and found a strong positive correlation between the values obtained via the two methods. Pearson’s correlation coefficient R² = 0.83. G: glucose limited; S: sulfate limited; P: phosphate limited.
(PDF)

S6 Fig Copy-number fluctuations of the plasmids monitored by qPCR in population samples over time. (**A**) Copy number of the plasmid as determined by qPCR using population DNA over time. Each color corresponds to a condition as described in panel B. (**B**) Average plasmid copy number for the high-copy and low-copy plasmid collections grown for 20 generations in glucose-limited, sulfate-limited, and phosphate-limited conditions.
(PDF)

S1 Table Strains and strain collections used in the study. (XLSX)

S2 Table Fitness measurements from the pooled competitions of the plasmid and deletion collections. Notes: 1) Name: name of the gene. 2) Collections: MM1N (haploid deletion); MM2N (heterozygous deletion); CEN (low-copy plasmid (MoBY-ORF)); 2micron: (high-copy plasmid (MoBY-ORF-v2)). 3) Example: MM1N-phosphate (average of the two fitness values for the haploid deletion collection competed in the phosphate-limited condition). 4) The number of replicates indicates the number of experiments for which the fitness was measured (maximum of 12 experiments).
(XLSX)

S3 Table Fitness measurements from the pooled competitions of the barcoder collection. Notes: 1) Barcode name. 2) Sequence of the barcode detected by barseq. 3) The limitation indicates the condition (glucose, sulfate, or phosphate limitation) in which a particular fitness was determined.
(XLSX)

S4 Table Identities, frequencies, and predicted effects of the mutations identified in experimental evolution studies. Notes: 1) Mutations detected by whole-genome sequencing of populations and single clones from previous evolution experiments performed in batch and continuous cultures. 2) The reference base was not always reported in the original studies. 3) The number of mutations refers to the number of samples in which the gene was found to be mutated. 4) Class indicates the class of mutations. 5) The sample corresponds to the sample name in the original papers. 6) The population frequency was reported when known. 7) Found in clone: in cases where both population and single clones were sequenced, we indicated whether the mutation was detected in both sample types. 8) Background: name of the strain used in the studies. 9) The reference indicates the papers in which the mutations were published (see the references at the end of the publication for a more detailed listing). 10) Snpeff: http://snpeff.sourceforge.net/. 11) Detrimental/Beneficial: if the mutation affected a gene for which a fitness decrease (detrimental) or increase (beneficial) had been measured in the same conditions, we reported the fitness effect of the mutation.
(XLSX)

S5 Table Beneficial mutations identified in the pooled competitions. Notes: 1) Systematic and Standard names correspond to the name of the target gene. 2) Only mutations with a fitness >0.10 are reported here. 3) The limitation indicates the condition in which a particular fitness was determined. 4) Detected in Evolution: if a mutation in the gene was reported in an evolution experiment, the SNPeff effect is reported; otherwise, “Not found” is indicated. 5) Recurrent: indicates the number of times the gene was found mutated in the evolution experiments. 6) The conditions in which the gene was been found mutated in the evolution experiments.
(XLSX)

S6 Table Beneficial mutations in the previous evolution experiments. Notes: 1) The number of beneficial mutations refers to the number of beneficial mutations per sample. 2) The conditions refer to the conditions used during the evolution experiment. 3) Other events: some of the studies reported copy-number variants or determined that the mutation was adaptive. 4) The mutation total refers to the number of mutations reported per sample. 5) Generations refer to the number of generations for which the sample was selected. 6) Ratio Benet total: the ratio of the number of beneficial mutations to the total number of mutations. 7) Ratio benef Generation: ratio of the number of beneficial mutations to the number of generations of selection.
(XLSX)

S7 Table Fitness measurements from pairwise competitions versus those from pooled competitions. Notes: 1) Comparison of the fitness of each strain between the pooled competitions (barseq average) and the pairwise competitions (individual average) performed in the same nutrient-limited condition. 2) Barcodes were verified by Sanger sequencing. 3) Mutations in *WHI2* are recurrently found in the yeast collections and are associated with a fitness increase in multiple conditions [79]. We verified the absence of mutations in *WHI2* by Sanger sequencing.
(XLSX)

S8 Table Primers used in this study. (XLSX)

S9 Table Barcode sequences from the collections used in this study. (XLSX)

S10 Table Summary statistics for barcode sequencing experiments. (XLSX)

S1 File Perl script for demultiplexing sequencing files. (PL)

S2 File C script used for barcode assignment. (C)

S3 File R script used for linear regression for fitness calculations. (R)
