# Supplementary figures and images for: High-Throughput Identification of Adaptive Mutations in Experimentally Evolved Yeast Populations
Source: PLoS Genet. 2016 Oct 11;12(10):e1006339. doi: 10.1371/journal.pgen.1006339 (PMC5065121; doi:10.1371/journal.pgen.1006339)

Figure S1

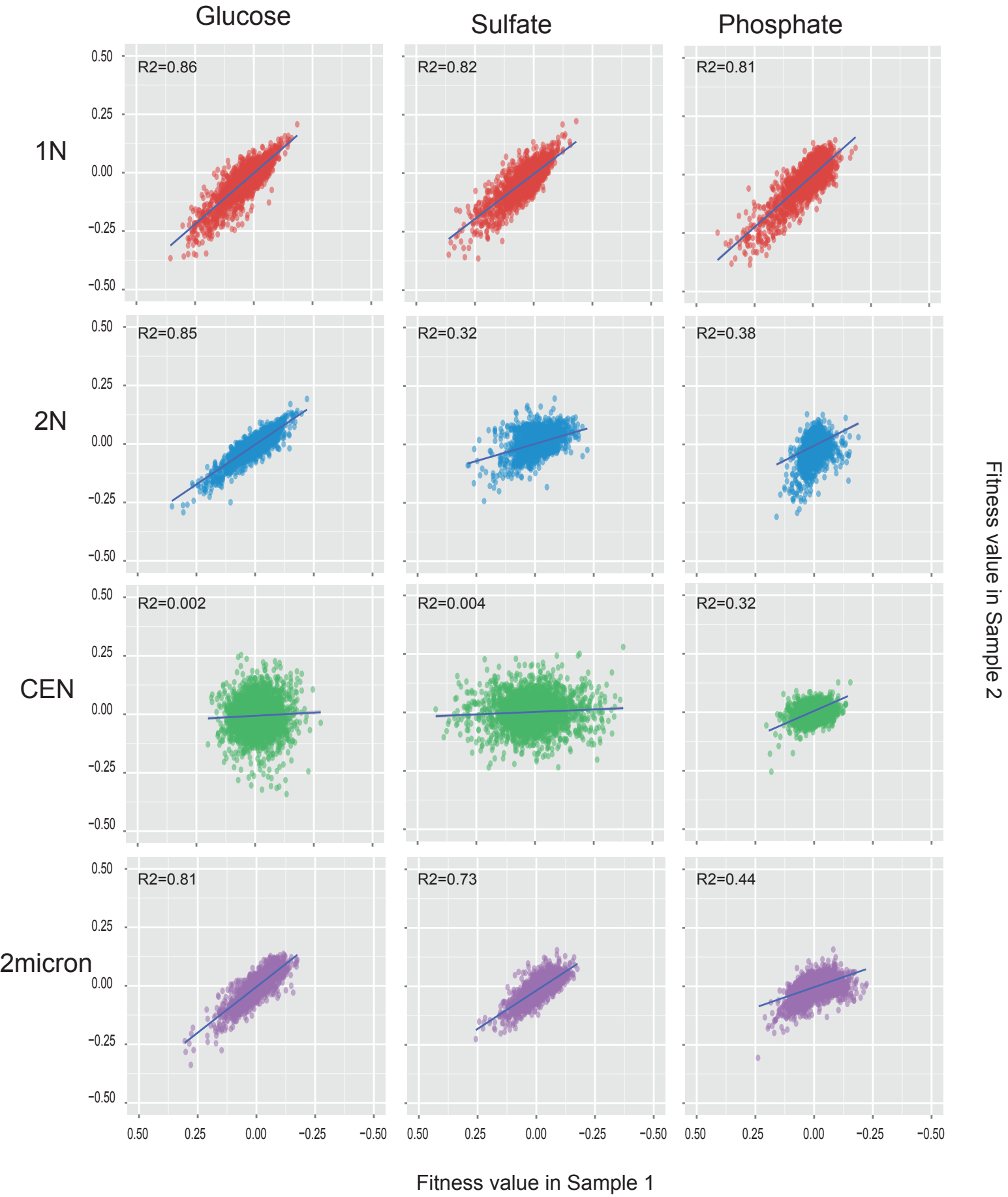

Supplement: S1 Fig — Each experiment is labeled with the condition (G, S, or P, for glucose, sulfate, or phosphate limitation) and the replicate (1 or 2). (PDF) [file pgen.1006339.s001.pdf]

Figure S2

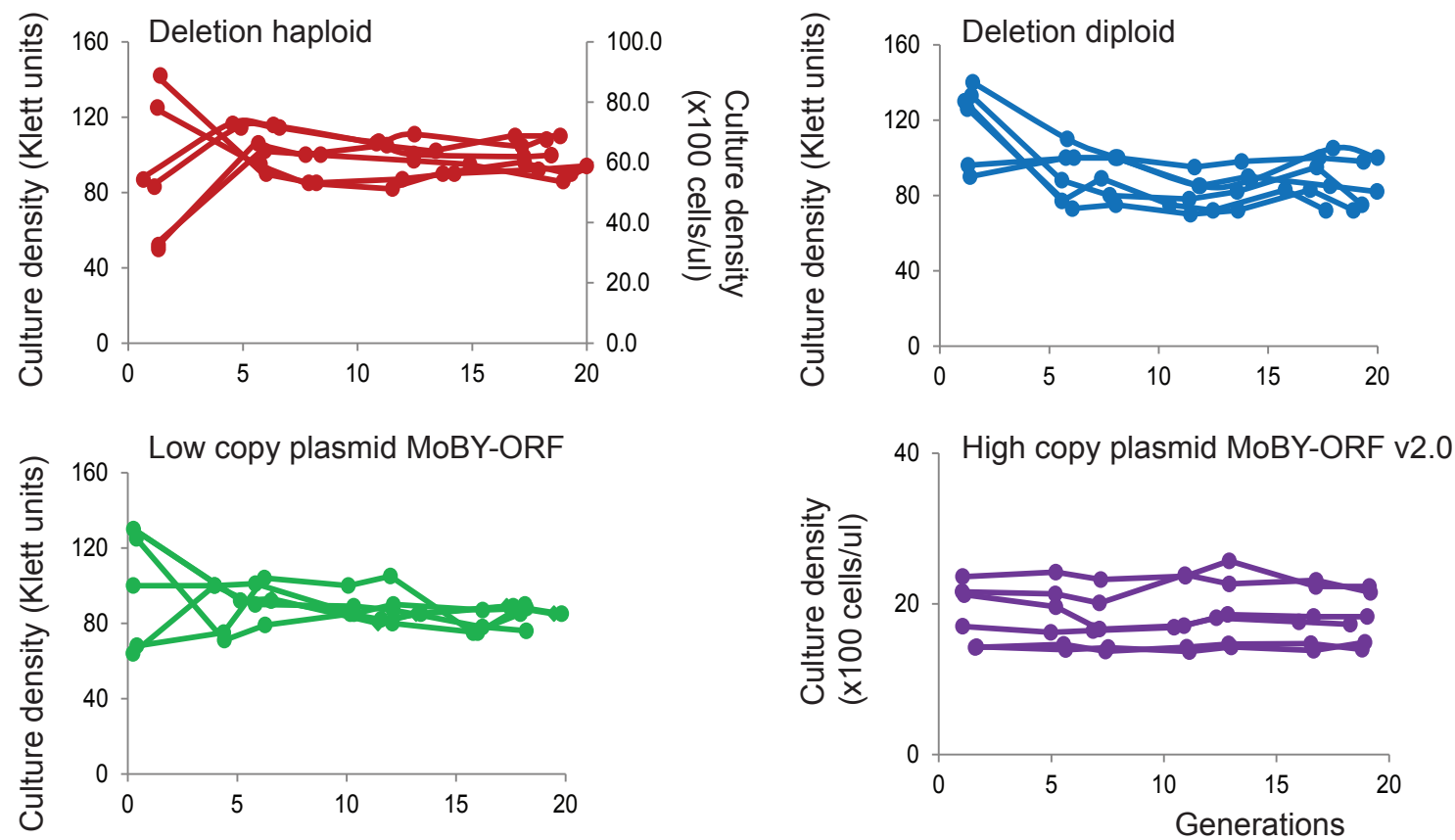

Supplement: S2 Fig — Cell density over time is shown for each pool grown in glucose, sulfate, and phosphate limitation for 20 generations. (PDF) [file pgen.1006339.s002.pdf]

Figure S4

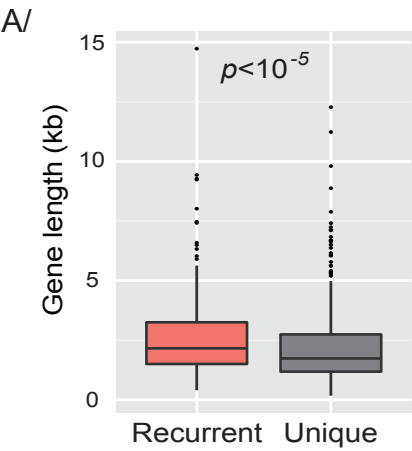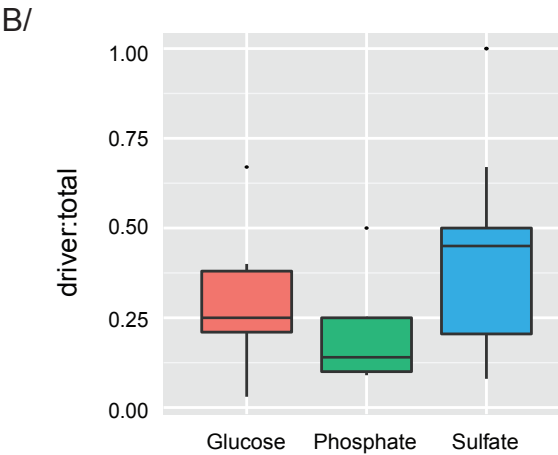

Supplement: S4 Fig — (A) Distribution of gene size for recurrently mutated genes and genes mutated in only sample, respectively. The significance of the difference between the two boxplots was estimated using a Wilcoxon rank-sum test. (B) The ratio of driver mutations to total mutations was not condition-specific (p = 0.61, 0.05, and 0.05 for glucose limitation, sulfate limitation, and phosphate limitation, respectively). (PDF) [file pgen.1006339.s004.pdf]

Figure S5

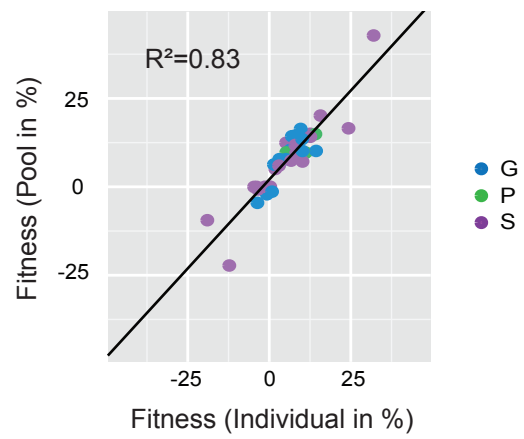

Supplement: S5 Fig — The fitness values in the pooled experiments are relative to the mean fitness of the population. We therefore compared the fitness of 51 strains measured in the pooled assays to that measured in pairwise fitness assays and found a strong positive correlation between the values obtained via the two methods. Pearson’s correlation coefficient R² = 0.83. G: glucose limited; S: sulfate limited; P: phosphate limited. (PDF) [file pgen.1006339.s005.pdf]

Figure S6

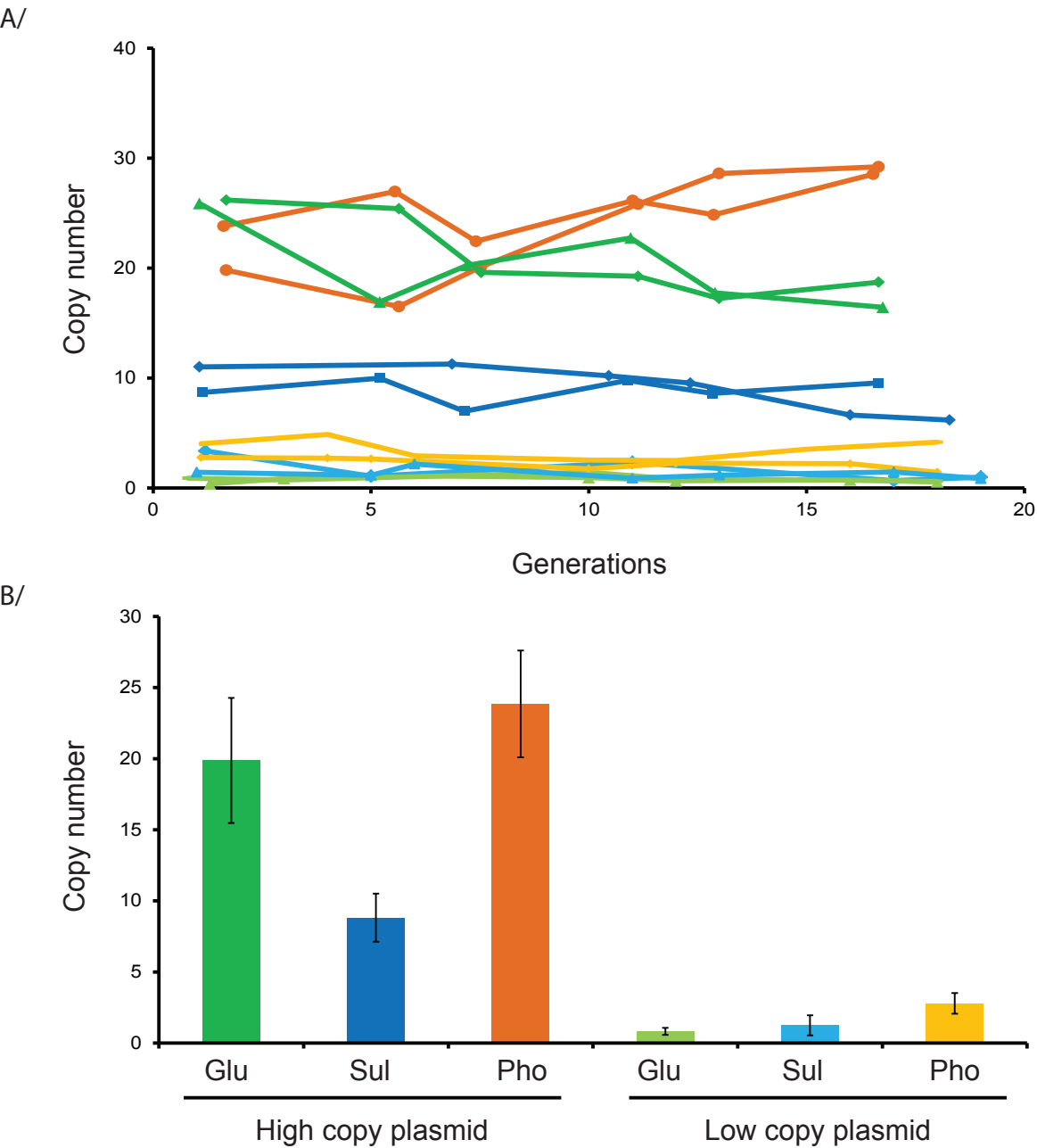

Supplement: S6 Fig — (A) Copy number of the plasmid as determined by qPCR using population DNA over time. Each color corresponds to a condition as described in panel B. (B) Average plasmid copy number for the high-copy and low-copy plasmid collections grown for 20 generations in glucose-limited, sulfate-limited, and phosphate-limited conditions. (PDF) [file pgen.1006339.s006.pdf]
