# Supplementary material for: High-Throughput Identification of Adaptive Mutations in Experimentally Evolved Yeast Populations
Source: PLoS Genet. 2016 Oct 11;12(10):e1006339. doi: 10.1371/journal.pgen.1006339 (PMC5065121; doi:10.1371/journal.pgen.1006339)
Supplement: S3 Fig — Each box, represents the relative frequency (log2 ratio of the frequency) of one strain over time. Each line (blue and red) represents the linear regression used to calculate the relative fitness between generations 6 and 20. (PDF) [file pgen.1006339.s003.pdf]

Figure S3

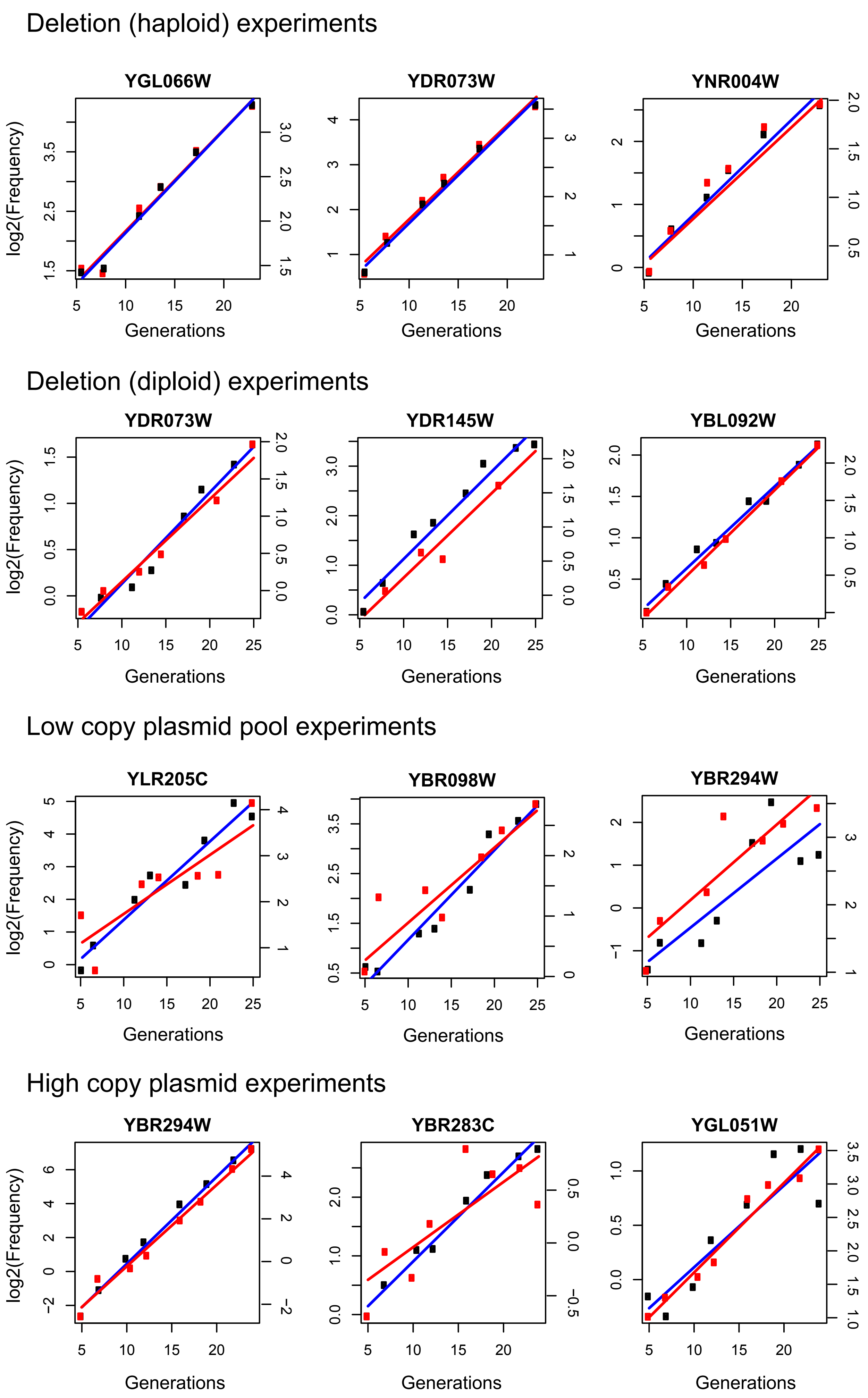
Deletion (haploid) experiments
YGL066W
YDR073W
YNR004W
Deletion (diploid) experiments
YDR073W
YDR145W
YBL092W
Low copy plasmid pool experiments
YLR205C
YBR098W
YBR294W
High copy plasmid experiments
YBR294W
YBR283C
YGL051W
log2(Frequency)
Generations
